# Healthcare utilization in Ghana: Insights from the 2017 Ghana Living Standard Survey

**DOI:** 10.1371/journal.pone.0306032

**Published:** 2024-06-25

**Authors:** Derek Anamaale Tuoyire, Leonard Baatiema, Duah Dwomoh, Samuel Bosomprah

**Affiliations:** 1 Department of Community Medicine, School of Medical Sciences, University of Cape Coast, Cape Coast, Ghana; 2 Department of Health Policy Planning and Management, School of Public Health, University of Ghana, Legon, Ghana; 3 Department of Biostatistics, School of Public Health, University of Ghana, Legon, Ghana; SDD - University of Business and Integrated Development Studies, GHANA

## Abstract

**Introduction:**

The persistence of healthcare utilization disparities in Ghana despite several policy efforts highlights the urgency of understanding its determinants to enhance equitable health access. We sought to examine the determinants of healthcare utilization in Ghana.

**Methods:**

We used the 2017 Ghana Living Standard Survey (GLSS) data. This was a cross-sectional design, which employed a stratified two-stage random sampling technique. We analyzed data involving 8,298 respondents with information on visits to healthcare facilities for services on account of illness or injury two weeks prior to the survey. Pearson’s chi-squared test was used to assess the distribution of healthcare utilization across background characteristics. Further, we used multivariable Poisson regression model with robust standard error to identify factors independently associated with healthcare utilization.

**Results:**

Among the 8,298, the median age was 24 years (interquartile range = 7–47), 45% were males, and 45% had no education. About 42% of respondents utilized health services during an episode of illness or injury. Age, sex, educational level, marital status, wealth quintile, health insurance and severity of illness/injury were independently associated with healthcare utilization. Healthcare utilization increased significantly with wealth quintiles—those in the highest wealth quintiles had about 22% increased utilization compared to those in the lowest wealth quintiles (aPR = 1.22; 95%CI = 1.13–1.32) while it was about 77% higher among those who had valid health insurance compared to those without (aPR = 1.77; 95% CI = 1.68–1.86). Regarding severity of illness or injury, those with severe conditions were about 65% more likely to utilize healthcare services compared to those with acute conditions (aPR = 1.65; 95% CI = 1.53–1.78).

**Conclusion:**

Our study underscores the importance of socio-economic factors and health insurance in healthcare utilization in Ghana. Addressing these can pave the way for more equitable access to healthcare services across all segments of the population.

## Introduction

Healthcare utilization disparities remains a significant challenge in Ghana’s health delivery system. As a crucial indicator of a nation’s health system efficiency, healthcare utilization reflects the intricate balance between availability and accessibility of healthcare for optimal population’s health outcomes [1]. While global strides in healthcare access and utilization have been significant, more than one billion people are unable to obtain essential healthcare service for various reasons, with low and middle-income countries (LMICs) like Ghana disproportionately affected [2, 3]. Ghana, like other LMICs continues to grapple with generally low healthcare utilization of about 1.1 outpatient visits per capita [4]. Moreover, there are existing disparities in healthcare service access and utilization in Ghana on account of gender, socio-economic status, rural-urban, insurance status, health state among others.

The persistence of healthcare utilization disparities in Ghana despite several policy efforts such as the implementation of the Community Health Planning and Services (CHPS) and National Health Insurance Scheme (NHIS) initiatives highlight the urgency of understanding its determinants to enhance equitable healthcare access. These disparities, left unaddressed, could exacerbate existing health inequalities in disease burden in the country. Prior studies on the subject have mainly focused on maternal and child healthcare utilization [5–7] or population segments such as the aged [8, 9], those with chronic health conditions [10], and those with health insurance subscription [11]. While these studies have provided some useful insights on the subject, their generalizability to the Ghanaian population is limited since they only represent specific segments of the population.

Sequel to the fragmented nature of prior studies in Ghana, the multifaceted determinants of healthcare utilization, from individual predispositions to institutional factors, remain inadequately understood in the country’s context. This knowledge gap impedes efficient policy formulation and resource allocation to address the complex disease burden in Ghana characterized by an increasing burden of noncommunicable diseases along with persistent communicable, maternal, perinatal and nutritional diseases [12, 13]. The decision to seek and utilize healthcare services often stems from the interplay of multiple factors, from individual predispositions to institutional factors. For instance, marital status, educational status, income, wealth, and health insurance have been found associated with healthcare utilization [14, 15].

We sought to examine the determinants of healthcare utilization in Ghana. By relying on nationally representative Ghana Living Standards Survey (GLSS) data, the study extends the current understanding of healthcare utilization in Ghana to the general population. This will provide insights and an opportunity for policy decision makers to design more targeted interventions towards bridging disparities in disease burden and foster a more equitable access to- and utilization of healthcare services [16].

## Methods

### Data source

The study relied on data from seventh rounds (2016/17) of the GLSS. The GLSS is Ghana’s customized edition of the Living Standards Measurement Study (LSMS) project which began in 1980 by the World Bank’s Policy Research Division for developing countries. The survey was based on a cross-sectional design, which employed a two-stage stratified random sampling technique. In the first stage, enumeration areas (i.e., primary sampling units (PSU)) were stratified into regions and rural-urban localities. The PSUs were then selected from each stratum using probability proportional to population size sampling technique. In the second stage, households were selected using systematic random sampling technique.

Trained field assistants collected data using structured questionnaire. The GLSS 2016/17 successfully surveyed 16,000 household from which 58,596 respondents were interviewed. Part of the data collected on health included information on visits to healthcare facilities for services on account of illness or injury two weeks prior to the survey. Thus, the final samples arrived at for the purpose of this current analysis was 8,298 respondents. The GLSS data is publicly available upon request from the Ghana Statistical Service (GSS). A data request form was downloaded from the website of the GSS, filled and submitted via email (datarequest@statsghana.gov.gh). The request was subsequently reviewed by the GSS and approval was granted to download the datasets from the following web link https://statsghana.gov.gh/gssdatadownloadspage.php.

### Variables

The primary outcome was healthcare utilization. This was measured based on whether a respondent visited a healthcare facility following an episode of illness or injury prior to the survey. For the purposes of analysis, those who visited a healthcare facility following an episode of illness or injury prior to the survey were considered to have utilized healthcare services and coded “1” while participants who did not were coded “0”.

Relevant background attributes of the respondents constituted the independent variables for the study. The selection of these variables was informed by the literature, particularly the Behavioural Model of Health Service Utilization developed by Roland M. Andersen in 1968 [17] Andersen posits that healthcare utilization is influenced by people’s predisposition to utilize healthcare services, certain impeding or enabling factors, as well as factors that elicit a relative need for care in health facilities [17, 18]. Hence, the independent variables considered were age, sex, educational level, and marital status, wealth status, residence, health insurance, severity of illness/injury, and disability status.

### Data analysis

Background characteristics were summarized using frequencies and proportions for categorical variables while median and interquartile range were used for continuous variables. Pearson’s chi-squared test was used to assess the distribution of healthcare utilization across background characteristics. We used multivariable Poisson regression model with robust standard error to identify factors independently associated with healthcare utilization. In building a parsimonious model, factors that were individually associated with healthcare utilization were candidate for inclusion in the multivariable models. Factors were removed at a p-value of less than 0.10. All analyses were performed using Stata 16 (StataCorp, College Station, TX, USA).

### Ethics statement

The GLSS data were fully anonymized and publicly available upon request from the Ghana Statistical Service (GSS). A data request form was downloaded from the website of the GSS, filled and submitted via email (datarequest@statsghana.gov.gh). The request was subsequently reviewed by the GSS, and approval was granted to download the datasets from the following web link https://statsghana.gov.gh/gssdatadownloadspage.php. We did not seek informed consent from the individuals included in this study because the data were fully anonymized and cannot be linked to any individual.

## Results

### Background characteristics and healthcare utilization

A total of 8,298 responders were included in the analysis. Among the 8,298, the median age was 24 years (interquartile range = 7–47), 45% were males, 45% had no education, and 35% were married or cohabiting (Table 1). They mostly resided in rural (69%) areas with 28% in the lowest wealth quintiles while 40% had valid Ghana health insurance card. With respect to their health, about 70% reported to have experienced an episode of acute illness or injury prior to the survey, while only about 4% reported having had one form of disability or the other. About 42% of respondents utilized health services during an episode of illness or injury, which varied by background characteristics including, age, birth sex, education, wealth quintiles, and severity of illness (Table 1).

**Table 1:** Percentage of respondents with healthcare utilization by background characteristics.

Characteristic	Respondents	Healthcare utilization	Chi2; p-value
	N (% of total)	n (%)	
*Age group*			
Median (IQR) 24(7–47)			
<20	3665 (44.2)	1678 (44.8)	44.9; P < 0.001
20–39	1891 (22.8)	741 (39.2)	
40–59	1576 (18.9)	585 (37.1)	
60+	1166 (14.1)	467 (40.0)	
*Sex*			
Male	3715 (44.8)	1460 (39.3)	17.7; P < 0.001
Female	4583 (55.2)	2011 (43.9)	
*Educational level*			
No Education	3727 (44.9)	1631 (43.8)	13.6; P < 0.001
Primary & JSS/JHS	3664 (44.2)	1451 (39.6)	
Secondary+	907 (10.9)	389 (42.9)	
*Marital status*			
Never/previously married	5425 (65.4)	2314 (42.7)	4.4; P = 0.036
Married/cohabiting	2873 (34.6)	1157 (40.3)	
*Residence*			
Rural	5687 (68.5)	2305 (40.5)	12.5; P < 0.001
Urban	2611 (31.5)	1166 (44.7)	
*Wealth quintile*			
Lowest	2337 (28.2)	894 (38.3)	21.6; P < 0.001
Low	1921 (23.2)	804 (41.9)	
Middle	1463 (17.6)	633 (43.3)	
High	1349 (16.3)	580 (43.0)	
Highest	1228 (14.8)	560 (45.6)	
*Health insurance*			
No	4960 (59.8)	1557 (31.4)	552.1; P < 0.001
Yes	3338 (40.2)	1914 (57.3)	
*Severity of illness/injury*			
Acute (1–3 days)	5818 (70.1)	2074 (35.6)	305.9; P < 0.001
Mild (4–7 days)	1794 (21.6)	1016 (56.6)	
Severe (8+ days)	686 (8.3)	381 (55.5)	
*Disability status*			
Non-PWD	7985 (96.2)	3347 (41.9)	0.7; P = 0.419
PWD	313 (3.8)	124 (39.6)	
**Total**	**8298 (100)**	**3471 (41.8)**	

### Factors independently associated with healthcare utilization

In a multivariable parsimonious Poison regression model, seven factors, namely; age, sex, educational level, marital status, wealth quintile, health insurance and severity of illness/injury were independently associated with healthcare utilization (Table 2). Healthcare utilization appeared significantly lower with each subsequent higher age group, ranging from 20% (adjusted prevalence ratio (aPR) = 0.80; 95% CI = 0.73–0.87) among those aged 20–39 years to 27% (aPR = 0.73; 95% CI = 0.67–0.79) among those aged 60 years and above. Healthcare utilization was about 11% higher among females compared to males (aPR = 1.11; 95% CI = 1.06–1.17). With reference to those without education, healthcare utilization was 7% (aPR = 0.93; 95% CI = 0.88–0.98) lower among those with primary/JSS/JHS education (Table 2).

**Table 2:** Factors independently associated with healthcare utilization. N = 8,298.

Factors	Crude Model		Adjusted Model		Wald Test	
	PR	95% CI	aPR	95% CI	Chi2	P-value
*Age group*						
<20	1.00	[1.00,1.00]	1.00	[1.00,1.00]	44.69	<0.001
20–39	0.86**	[0.80,0.91]	0.80**	[0.73,0.87]		
40–59	0.81**	[0.75,0.87]	0.74**	[0.67,0.80]		
60+	0.87**	[0.81,0.95]	0.73**	[0.67,0.79]		
*Sex*						
Male	1.00	[1.00,1.00]	1.00	[1.00,1.00]	17.48	<0.001
Female	1.12**	[1.06,1.18]	1.11**	[1.06,1.17]		
*Educational level*						
No Education	1.00	[1.00,1.00]	1.00	[1.00,1.00]	13.49	<0.001
Primary & JSS/JHS	0.90**	[0.86,0.96]	0.93**	[0.88,0.98]		
Secondary+	0.98	[0.90,1.07]	1.02	[0.94,1.12]		
*Marital status*						
Never/previously married	1.00	[1.00,1.00]	1.00	[1.00,1.00]	4.32	0.037
Married/cohabiting	0.94*	[0.89,1.00]	1.13**	[1.05,1.21]		
*Residence*						
Rural	1.00	[1.00,1.00]	-	-		
Urban	1.10**	[1.04,1.16]	-	-		
*Wealth quintile*						
Lowest	1.00	[1.00,1.00]	1.00	[1.00,1.00]	21.16	<0.001
Low	1.09*	[1.02,1.18]	1.10**	[1.03,1.18]		
Middle	1.13**	[1.05,1.22]	1.12**	[1.04,1.21]		
High	1.12**	[1.04,1.22]	1.11**	[1.03,1.20]		
Highest	1.19**	[1.10,1.29]	1.22**	[1.13,1.32]		
*Health insurance*						
No	1.00	[1.00,1.00]	1.00	[1.00,1.00]	546.96	<0.001
Yes	1.83**	[1.74,1.92]	1.77**	[1.68,1.86]		
*Severity of illness/injury*						
Acute (1–3 days)	1.00	[1.00,1.00]	1.00	[1.00,1.00]	336.53	<0.001
Mild (4–7 days)	1.59**	[1.51,1.68]	1.58**	[1.50,1.67]		
Severe (8+ days)	1.56**	[1.44,1.68]	1.65**	[1.53,1.78]		
*Disability status*						
Non-PWD	1.00	[1.00,1.00]	-	-		
PWD	0.95	[0.82,1.09]	-	-		

Model parameters were estimated using Poisson regression with robust standard error; Abbreviations: PR = prevalence ratio; aPR = adjusted prevalence ratio; CI = confidence intervals; JSS = Junior Secondary School; JHS = Junior High School; PWD = Persons with Disability. P-values for each category of factors: p < .10,

* p < .05,

** p < .01

Healthcare utilization increased significantly with wealth quintiles—those in the highest wealth quintiles had about 22% increased utilization compared to those in the lowest wealth quintiles (aPR = 1.22; 95%CI = 1.13–1.32); while it was about 77% higher among those who had valid health insurance compared to those without (aPR = 1.77; 95% CI = 1.68–1.86). Regarding severity of illness or injury, those with severe conditions were about 65% more likely to utilize healthcare services compared to those with acute conditions (aPR = 1.65; 95% CI = 1.53–1.78) (Table 2).

## Discussion

Our study examined the factors independently associated with healthcare utilization in Ghana using nationally representative GLSS data. Among the 8,298 respondents, the median age was 24 years, with an equal representation of males and those without formal education, both at 45%. Interestingly, only 42% sought healthcare services during an episode of illness or injury in the last two weeks prior to the survey. The data revealed a discernible inverse relationship between age and healthcare service use, with older age brackets showing reduced utilization rates, specifically by 20% for those aged 20–39 and 27% for those 60 and above. Women were more likely to access healthcare, with an 11% higher utilization rate than men, while individuals with primary to JSS/JHS educational levels had a 7% lesser rate compared to the uneducated. Furthermore, a significant association between wealth and healthcare use emerged, as those in the highest wealth brackets exhibited a 22% increased usage. Additionally, the presence of valid health insurance amplified healthcare utilization by a notable 77%. Lastly, individuals confronted with more severe health challenges were 65% more likely to turn to professional healthcare than those with milder conditions.

The age-related decline in healthcare utilization, especially among those aged 60 years and above, contrasts with results from other studies [3, 9, 19–21] where older population tend to access healthcare more frequently, often due to chronic conditions. This discrepancy may suggest economic, cultural, or healthcare accessibility factors. However, our finding also compared with other studies [22, 23]. The increased use of healthcare among women, as observed in our study, reflects global trends where women seek medical care more often due to reproductive health needs [24] and have a generally high health-seeking behaviour [3, 25, 26]. Our finding that education, albeit at primary to JSS/JHS levels, was associated with reduced healthcare use is counterintuitive because several studies in different settings have typically found that higher education is associated with increased health awareness and healthcare utilization [3, 8, 9]. This could be due to low health literacy or knowledge about the importance of utilizing healthcare services [27] and may warrant a further study into the Ghanaian educational content or societal dynamics around health.

Notably, the role of wealth in enabling healthcare access is a common trend, with the economically advantaged often having better healthcare accessibility, as observed in many studies from both developed and developing country settings [25, 28–30]. The stark difference in healthcare utilization due to health insurance membership [11, 25, 28] in Ghana underscores the transformative potential of health insurance in low- and middle-income countries. Health insurance schemes have been identified as important strategies for attaining universal health coverage by providing equitable access to essential health services and reducing financial barriers to healthcare utilization [28, 31, 32]. The association between the severity of health conditions and increased healthcare utilization is intuitive and aligns with global observations that individuals with severe symptoms are more likely to seek medical care. As Andersen [1995] posited and corroborated by prior studies [18, 24, 33], one’s evaluation of the severity of their illness determines their motivation. While several of our findings align with general trends, unique deviations especially related to age and education emphasize the importance of localized studies and solutions.

The strengths and limitations of our study provides valuable insight into the scope and reliability of our findings. One of the most significant strengths is the use of the nationally representative survey data, which is credible due to its robust methodological design. Additionally, the large number of respondents lends statistical power to the findings and minimizes margin of error. However, we also have some limitations worth acknowledging. First, the cross-sectional design suggests that the data is a snapshot in time, potentially missing temporal trends, and dynamic changes in health seeking behaviours. Second, while our study does account for several determinants of healthcare utilization, there exists unmeasured variables including chronic disease status of individuals, distance to health facility and perceptions about quality of care or other external factors that might influence the results. Finally, the focus on individuals who had information on visits due to illness or injury may introduce a selection bias. Balancing these strengths and limitations, the study offers a robust overview of healthcare utilization in Ghana, while still leaving room for deeper analysis of the subject, including possibly conducting qualitative evaluations to further understand the intricacies of our findings.

Our findings have implications for the healthcare landscape in Ghana. With the marked increase in healthcare utilization among the insured, it suggests that policy interventions aimed at broadening health insurance coverage could potentially improve health outcomes and equity. The age variation in healthcare utilization highlights the need for strategies tailored to different age brackets. Older populations, which showed a decline in healthcare use, might benefit from targeted interventions. Our study illuminates several options for policymakers, healthcare providers, and community leaders to intervene, ensuring more inclusive and effective health service delivery.

## Conclusions

Our study underscores the multifaceted determinants of healthcare utilization in the Ghanaian population. Factors like age, gender, education, economic status, and health insurance coverage interplay to shape healthcare decisions. Overall, the study emphasizes the need for targeted, equity-focused strategies to ensure comprehensive healthcare access for all Ghanaians.
